# Dynamic brain glymphatic changes and cognitive function in COVID-19 recovered patients: a DTI-ALPS prospective cohort study

**DOI:** 10.3389/fpsyg.2025.1465660

**Published:** 2025-04-22

**Authors:** Chengcheng He, Jinmei Xie, Weiwei Fang, Baoqin Guo, Yangyang Shi, Anan Li, Hao Liu, Zhimin Zhu, Wenrui Bao, Xuan Niu, Shaoyu Wang, Juan Fu, Hua Li, Wenjuan Xie

**Affiliations:** ^1^Department of Medical Imaging, Yulin Hospital, The First Affiliated Hospital of Xi'an Jiaotong University, Yulin, China; ^2^Department of Medical Imaging, Xinyuan Hospital of Yulin, Yulin, China; ^3^School of Future Technology, Xi’an Jiaotong University, Xi'an, China; ^4^MR Research Collaboration, Siemens Healthineers, Shanghai, China; ^5^Department of Medical Imaging, The First Hospital Of Yulin, Yulin, China

**Keywords:** COVID-19 recovered patients, brain glymphatic function, DTI-ALPS, cognitive function, fatigue

## Abstract

**Objective:**

This study aimed to evaluate brain glymphatic function in COVID-19 recovered patients using the non-invasive Diffusion Tensor Imaging-Analysis Along the Perivascular Space (DTI-ALPS) technique. The DTI-ALPS technique was employed to investigate changes in brain glymphatic function in these patients and explore correlations with cognitive function and fatigue.

**Materials and methods:**

Follow-up assessments were conducted at 1, 3, and 12 months post-recovery. A total of 31 patients completed follow-ups at all three time points, with 30 healthy controls (HCs) for comparison.

**Results:**

Compared to HCs, COVID-19 recovered patients showed a significant decline in MoCA scores at 3 months post-recovery (*p* < 0.05), which returned to near-normal levels by 12 months. Mental fatigue, measured by the Fatigue Assessment Scale (FAS), was significantly higher in COVID-19 patients at all follow-up points compared to HCs (*p* < 0.05). The DTI-ALPS index in both hemispheres showed significant differences at 3 months post-recovery compared to HCs (*p* < 0.001), indicating increased glymphatic activity. Longitudinal analysis revealed a peak in the DTI-ALPS index at 3 months post-recovery, which then decreased by 12 months. Correlation analysis showed a significant negative correlation between the Bilateral brain hemisphere DTI-ALPS index and MoCA scores (right side: *r* = −0.373, *p* = 0.003; left side: *r* = −0.255, *p* = 0.047), and a positive correlation with mental fatigue (right side: *r* = 0.275, *p* = 0.032; left side: *r* = 0.317, *p* = 0.013).

**Conclusion:**

This study demonstrates dynamic changes in brain glymphatic function in COVID-19 recovered patients, with a peak in activity at 3 months post-recovery. These changes are associated with cognitive function and mental fatigue, suggesting potential targets for addressing neurological symptoms of long COVID. The non-invasive DTI-ALPS technique proves to be a valuable tool for assessing brain glymphatic function in this population.

## 1 Introduction

COVID-19, caused by the novel coronavirus, is a severe emerging respiratory viral infectious disease (Haider et al., 2020). Beyond the acute infection symptoms, long-term symptoms associated with COVID-19 have become a notable global public health issue (Soriano et al., 2022; Chow et al., 2023). While the novel coronavirus primarily affects the pulmonary respiratory system, leading to severe pneumonia, it also exhibits neurotropic properties that can cause damage to the brain (Xu et al., 2024; Reis et al., 2022). Many patients who experienced acute COVID-19 continue to exhibit significant fatigue and cognitive impairment long after recovery (Greene et al., 2024). Approximately 80% of hospitalized patients display sequelae post-recovery (Drouin et al., 2024).

The brain glymphatic system, consisting of perivascular spaces formed by astrocyte endfeet and vascular walls, functions similarly to the lymphatic system in clearing cellular metabolic waste (Mestre et al., 2020; Hablitz and Nedergaard, 2021). Recent studies indicate that 50% of COVID-19 patients exhibit blood–brain barrier disruption and elevated inflammatory cytokines in cerebrospinal fluid (Jarius et al., 2022). Patients with COVID-19-related neurological manifestations present with different cerebrospinal fluid characteristics (Jarius et al., 2022). Some studies suggest that neurological syndromes may result from SARS-CoV-2 infection leading to a reduction in olfactory sensory neurons and decreased cerebrospinal fluid outflow, subsequently causing glymphatic circulation disturbances (Wostyn, 2021; Kempuraj et al., 2023). Over time, this can lead to the accumulation of toxic substances in the central nervous system (Kempuraj et al., 2023).However, these studies primarily focus on the chemical environment of cerebrospinal fluid and have not extensively examined the relationship between cognitive function changes and glymphatic function post-COVID-19 infection. If confirmed, glymphatic system function could potentially become a target for addressing the sequelae of COVID-19.

As early as 2012, Iliff et al. discovered, using fluorescence labeling techniques with two-photon microscopy, that perivascular spaces formed between astrocyte endfeet and vascular walls in the mouse brain have a function in clearing cellular metabolic waste (Iliff et al., 2012). They named this system the brain glymphatic system, demonstrating the existence of a lymphatic-like structure in the central nervous system. Subsequently, neuroimaging techniques such as dynamic contrast-enhanced MRI, PET and intrathecal gadolinium injection, were developed to assess the lymphatic system *in vivo* (Ding et al., 2021; Zhang et al., 2021; Schubert et al., 2019). However, these methods are invasive and can cause significant ionizing radiation exposure (Pei et al., 2024).In contrast, Diffusion Tensor Imaging-Analysis Along the Perivascular Space (DTI-ALPS) allows for non-invasive assessment of the glymphatic system using diffusion tensor imaging (Wang et al., 2023). Currently, studies using DTI-ALPS technology to investigate changes in brain glymphatic function in post-recovery COVID-19 patients are limited.

Therefore, we hypothesize that DTI-ALPS could be used to evaluate glymphatic changes post Covid-19, and that these changes gradually improve over time following recovery. To test this hypothesis, we employed DTI-ALPS technology to quantitatively assess brain glymphatic function in recovered COVID-19 patients, comparing glymphatic function at different time points, and analyzing the correlation between glymphatic function and cognitive performance.

## 2 Materials and methods

### 2.1 Participants

From January 2023 to March 2024, a prospective study was conducted at Xi’an Jiaotong University First Affiliated Hospital Yulin Hospital. Data were collected from 54 COVID-19 patients 1 month after testing negative. Follow-up was completed for 32 patients at 3 months and 31 patients at 1 year post-negative conversion, with one patient excluded due to incomplete imaging segmentation. Additionally, 30 healthy individuals, matched for age, sex, and education level, were included as the healthy control group (HCs). HCs were collected only once, with no longitudinal studies conducted. All participants were tested for COVID-19 using RT-PCR technology at the hospital.

All participants, including both COVID-19 patients and HCs, were required to complete a series of neuropsychological assessments, including the Montreal Cognitive Assessment (MoCA), Trail Making Test (TMT), Auditory Verbal Learning Test (AVLT), Digit Span Test (DST), and Fatigue Assessment Scale (FAS).

Inclusion Criteria was: Patients who met the diagnostic criteria for COVID-19 and tested negative after recovery (Atzrodt et al., 2020). Patients who voluntarily participated in the study and completed questionnaires and brain MRI follow-up. Aged 23–65 years, right-handed. Normal intelligence with at least a junior high school education. No history of psychiatric disorders. No psychological treatment post-recovery and no use of psychiatric medications in the past 2–3 weeks. All the people included had no basic diseases, drug use and healthy lifestyle. Voluntarily signed an informed consent form.

Exclusion Criteria was: Contraindications for MRI, such as the presence of metal implants. Poor MRI image quality, unsuitable for analysis. History of severe cardiovascular or cerebrovascular diseases, psychiatric or other physical illnesses. History of alcohol or substance dependence. Patients unable to complete follow-up.

This study was approved by the Ethics Committee of the First Affiliated Hospital of Xi’an Jiaotong University, with ethical approval granted in March 2022. The study was conducted in accordance with the World Medical Association’s Declaration of Helsinki. All participants provided written informed consent to participate in this study.

### 2.2 MRI acquisition parameters

Imaging data for all participants were acquired using a 3.0 T MRI scanner (MAGNETOM Spectra, Siemens Healthineers, Erlangen, Germany) with a head/neck coil. Participants were scanned in the supine position, with foam pads used to minimize movement during the scan.The MRI scanning parameters about DTI was: repetition time = 8,500 ms, echo time = 98 ms, field of view = 220 mm × 220 mm, matrix = 128 × 128, slice thickness = 4 mm, number of slices = 25, b = 0, 3,000 s/mm^2^ and 64 different diffusion encoding directions (Xiong et al., 2024; Georgiopoulos et al., 2024).

### 2.3 Data processing

**Figure 1:**
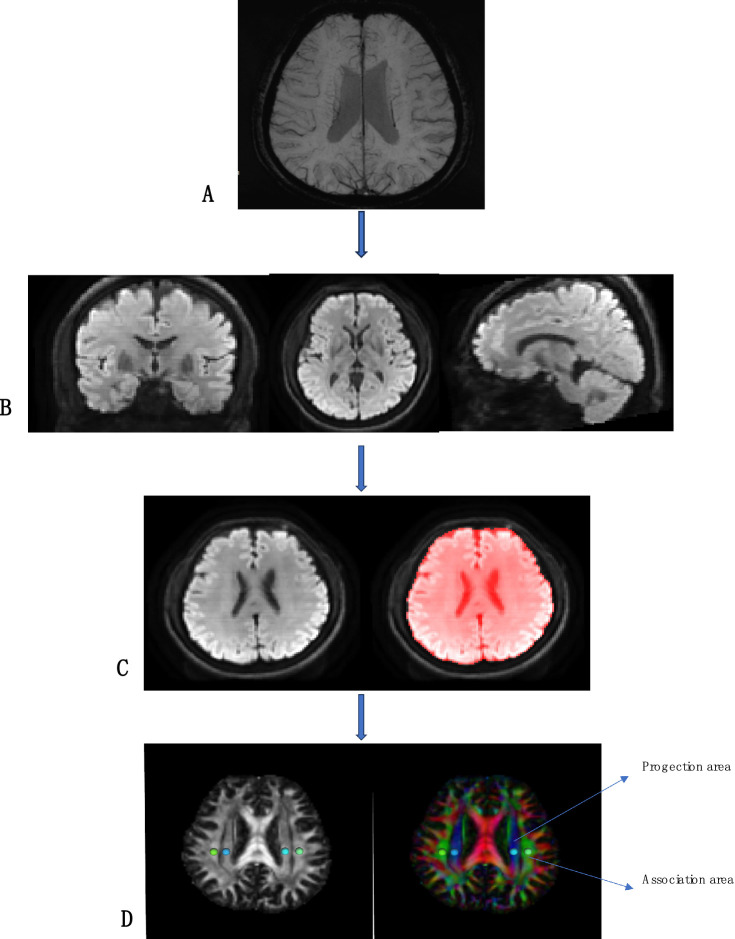
Flowcharts for DSI image processing. **(A)** SWI image shows medullary vein; **(B)** Adjust the orientation of the lateral ventricle to better show the projection fibers and association fibers; **(C)** Brain extraction; **(D)** DTIfa image and the DTI color map shows the direction of projected fibers (z-axis, blue) and joint fibers (y-axis, green). Place two regions of interest (ROI) to measure the double ROI utilization of projection (projection) and associated (correlation) fibers.

Post-processing was performed using DSI Studio software (version chen build 13 January 2022, http://dsi-studio.labsolver.org). The raw DTI sequence images were processed to obtain color-coded fractional anisotropy (FA) maps and diffusion rate images (Dx, Dy, Dz) for the X, Y, and Z axes, which are orthogonal to each other. ROIs were placed at the horizontal plane of the lateral ventricle body. Referring to phase images obtained from SWI post-processing, a circular region of interest (ROI) with a volume of 96 mm^3^ was selected in the blue and green areas of the color FA map, where medullary veins run perpendicularly to the lateral ventricles. ROIs were drawn in the projection fiber area and association fiber area on one side, and diffusion rate values in the X, Y, and Z directions were read from the three diffusion rate images (Refer to Figure 1 for the detailed process).

The ALPS index was calculated using the formula proposed by Taoka et al. It is defined as the ratio of the mean diffusivity (MD) in the projection fiber area on the X-axis (Dxx-Proj) and association fiber area (Dxx-Assoc) to the MD in the projection fiber area on the Y-axis (Dyy-Proj) and association fiber area on the Z-axis (Dzz-Assoc), as follows:


$$\begin{array}{l} DTI-\mathrm{ALPS index}=\mathrm{mean}\;\left(Dxx-\mathrm{proj,Dxx}-\mathrm{assoc}\right)/\\ \mathrm{mean}\;\left(Dyy-\mathrm{proj,Dzz}-\mathrm{assoc}\right) \end{array}$$

### 2.4 Statistical analysis

Clinical and imaging data were analyzed using SPSS 26.0. Depending on the data distribution type, quantitative data following a normal distribution were expressed as mean ± standard deviation, while non-normally distributed data were expressed as median. Categorical data were represented as numbers and percentages. Comparisons between groups were made using independent sample t-tests, Wilcoxon Mann–Whitney U tests, or chi-square tests. One-way repeated measures ANOVA or Friedman M tests were used to analyze cognitive scales and DTI-ALPS index differences across different time points, multiple comparisons were then performed. Pearson or Spearman correlation analysis was performed according to data distribution. A *p*-value <0.05 was considered statistically significant.

## 3 Results

### 3.1 Participant characteristics and clinical symptoms

In the cross-sectional comparison of general data, clinical characteristics and neurocognitive test results of patients at 1 month, 3 months, and 12 months follow-up are shown in Table 1. There were no statistical differences between the healthy control group and the three patient groups in terms of age, sex, years of education, height, weight, and BMI.

**Table 1:** Basic Information, neurocognition, and fatigue test scale for follow-up 1 months, follow-up 3 months, follow-up 12 months, and HCs groups.

Characteristics	Follow-up 1 month (*n* = 31)	Follow-up 3 months (*n* = 31)	Follow-up 12 months (*n* = 31)	HCs (n = 30)		t1/*χ*^2^ /Z1	*p*1	t2/*χ*^2^ /Z2	*p*2	t3/*χ*^2^ /Z3	*p*3
Age	41.23 ± 6.00	--	--	40.3 ± 10.3		0.442	0.661	--	--	--	--
Sex (male/female)	10/21	--	--	12/18		0.396	0.529	--	--	--	--
Education/years	17 (16, 18.5)	--	--	16 (16, 17)		−1.540	0.124	--	--	--	--
BMI	24.6 ± 2.7	24.6 ± 3.0	24.3 ± 3.0	23.4 ± 2.1		1.701	0.094	1.678	0.099	1.304	0.197
Overall cognitive function											
MOCA scale	28 (26.0, 30)	20 (19, 22)	30 (26, 30)	27.6 ± 2.6	28 (26, 30)	−0.508	0.611	−6.708	0.000	−1.456	0.145
Performing function											
STT-A (s)	32.3 ± 12.0	30 (28, 38)	31 (24, 48)	39.1 ± 15.3	37.1 (25.8, 52.0)	−1.938	0.057	−1.495	0.135	−0.729	0.466
STT-B (s)	90.0 (68, 115)	85 (67, 95)	97 (63, 111)	86.5 ± 71.2	83 (44, 97)	−1.587	0.112	−0.491	0.624	−1.667	0.096
Cognitive memory											
DST	15 (13, 15)	15 (12, 18)	14.5 ± 3.6	14.7 ± 2.6	14 (13, 17)	−0.373	0.709	−0.015	0.988	−0.227	0.821
Recitation in sequence	9 (8, 9)	8.9 ± 1.64	8.5 ± 2.1	8.8 ± 1.6	9 (8, 10)	−0.149	0.882	0.248	0.805	−0.666	0.508
Reverse recitation	5.58 ± 1.59	5 (4, 8)	6 ± 1.8	5.9 ± 1.5	6 (4, 7.0)	−0.712	0.479	−0.227	0.820	0.315	0.754
Auditory Word Test 1/2 (AVLT)											
Correct number of first 3 free recollections	26.2 ± 5.9	29 (25, 33)	31 (21, 34)	24.8 ± 7.3	26 (21, 30)	−0.797	0.429	−0.8420	0.065	−2.328	0.020
Short term delayed memory	11 (9, 11)	10 (10, 12)	10 (7, 11)	9.6 ± 2.9	9 (8, 12)	−1.274	0.203	−1.781	0.075	−0.524	0.600
Long term delayed memory	11 (9, 12)	11 (9, 12)	9 (6, 11)	9.2 ± 2.0	9 (8, 11)	−2.578	0.010	−2.197	0.028	−0.495	0.620
Recognition	22 (21, 24)	23 (21, 24)	22 (11, 24)	21.6 ± 3.8	23 (21, 24)	−0.854	0.393	−0.375	0.708	−1.640	0.101
Fatigue Rating Scale (FAS)	21 (18, 27)	20 (16, 25)	19 (15, 22)	17.1 ± 5.5	16 (14, 19)	−3.813	0.000	−2.821	0.005	−2.021	0.043
Physical fatigue	9 (7, 14)	9 (6, 13)	9 (5, 12)	9.3 ± 3.3	9 (6, 12)	−0.865	0.387	−0.334	0.738	−1.036	0.300
Mental fatigue	13 (10, 15)	12 (9, 12)	10 (9, 12)	7.8 ± 3.0	7.5 (5.8, 9.0)	−5.020	0.000	−4.780	0.000	−5.053	0.000

*p1: Comparison between the HCs group and the Follow-up 1 Month group; p2: Comparison between the HCs group and the Follow-up 3 Month group; p3: Comparison between the HCs group and the Follow-up 1 Month group. STT-A Trail Making Test A; STT-B: Trail Making Test B; p values less than 0.05 indicate statistical significance.*

In terms of overall cognitive function, the MoCA scores showed a significant decline only at 3 months post-recovery compared to the healthy control group (*p* < 0.01). However, the results of executive function tests (including STT-A and STT-B) did not show significant changes between different time points. For cognition and memory, the results of the digit span test, both forward and backward, did not show significant changes between different time points. However, in the AVLT test, long-delay recall showed a significant increase at the follow-up 1 and 3 motnes follow-up (*p* < 0.01), the total correct number of free recalls in the first three trials showed differences at the 12-month follow-up (*p* < 0.05). The Fatigue Assessment Scale (FAS) indicated that mental fatigue was significantly higher in patients at 1 month, 3 months, and 12 months post-recovery compared to healthy controls (*p* < 0.05) (Table 1).

In the longitudinal comparison, for overall cognitive function, the MoCA scores were significantly lower at the 3-month follow-up compared to the 1-month and 12-month follow-ups (*p* < 0.01). There were no significant differences in executive function. For cognitive and memory functions, short-delay recall and long-delay recall were significantly lower at the 12-month follow-up compared to the 3-month follow-up (*p* < 0.01). The FAS results showed that fatigue was significantly lower at the 12-month follow-up compared to the 3-month follow-up (*p* < 0.05) (Table 2).

**Table 2:** Neurocognitive and fatigue assessment measures at 1-month, 3-month, and 12-month follow-ups.

Characteristics	Follow-up 1 month (*n* = 31)	Follow-up 3 months (*n* = 31)	Follow-up 12 months (*n* = 31)	*χ* ^2^	*p*
Overall cognitive function					
MOCA scale	28 (26,30)	20 (19, 22)^#^	30 (26,30)◻	48.894	0.000
Performing function					
STT-A (s)	31 (27, 39)	30 (28, 38)	31 (24, 48)	1.480	0.477
STT-B (s)	90.0 (68, 115)	85 (67, 95)	97 (63, 111)	1.806	0.405
Cognitive memory					
Digital breadth test (DST)	15 (13, 15)	15 (12, 18)	14 (12, 17)	0.389	0.823
Recitation in sequence	9 (8, 9)	9 (8, 10)	8 (7, 10)	1.813	0.404
Reverse recitation	6 (4, 7)	5 (4, 8)	6 (5, 7)	0.173	0.917
Auditory Word Test 1/2 (AVLT)					
Correct number of first 3 free recollections	27 (23, 30)	29 (25, 33)	31 (21, 34)	2.644	0.267
Short term delayed memory	11 (9, 11)	10 (10, 12)	10 (7, 11)*⚪	9.321	0.009
Long term delayed memory	11 (9, 12)	11 (9, 12)	9 (6, 11)*⚪	15.600	0.000
Recognition	22 (21, 24)	23 (21, 24)	22 (11, 24)	4.252	0.119
Fatigue Rating Scale (FAS)	21 (18, 27)	20 (16, 25)	19 (15, 22)*	6.717	0.035
Physical fatigue	9 (7, 14)	9 (6, 13)	9 (5, 12)	5.523	0.063
Mental fatigue	13 (10, 15)	12 (9, 12)	10 (9, 12)	6.383	0.041

Compared with follow-up 1 month, *p < 0.05, ^#^*p* < 0.01; Compared with Follow-up 3 months, ⚪*p* < 0.05, ◻*p* < 0.01.

### 3.2 DTI-ALPS index analysis

To evaluate the reliability of the DTI-ALPS index, radiologists who were blinded to the participants’ group allocation (i.e., COVID-19 patients or healthy controls) and clinical information placed regions of interest (ROIs) for each participant and repeated the placement after 3 days. The intraclass correlation coefficient (ICC) was calculated, and the mean value was obtained. In this study, the ICC for the defined ROIs was 0.886 (*p* < 0.01), indicating excellent reliability.

**Figure 2:**
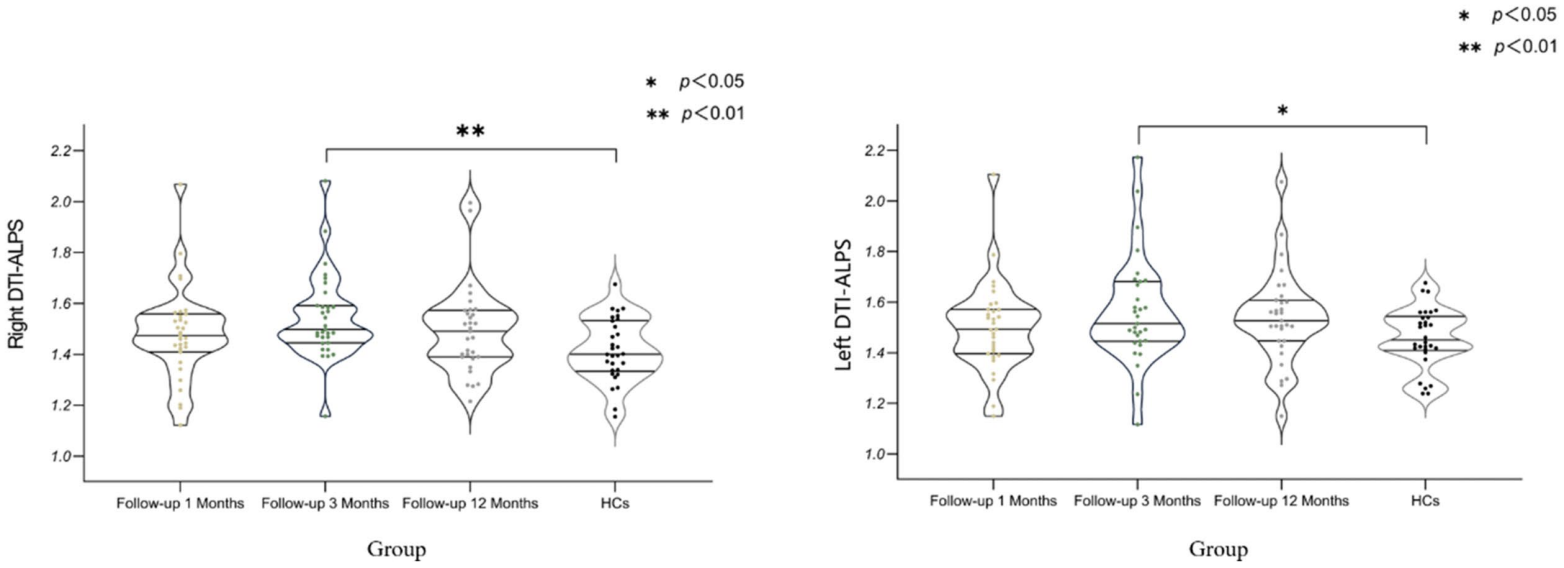
Differences in DTI-ALPS Index Between COVID-19 Recovery Patients at Different Stages and Healthy Controls. DTI-ALPS: diffusion tensor image analysis along the perivascular space; HCs: healthy controls (Data for healthy controls (HCs) were collected at a single time point, with no longitudinal follow-up).

Table 3 and Figure 2 shows the differences 0ndex between COVID-19 convalescent patients and healthy controls at different time points. The differences in the left and right brain measurements at the 3-month follow-up were statistically significant compared to the control group (*p* < 0.001), while the differences at the 1-month and 12-month follow-ups were not statistically significant (*p* > 0.05). This table demonstrates the differences in Brain lymphatic circulation function between COVID-19 patients and healthy controls at different follow-up periods, highlighting the significant changes that occurred at the 3-month mark.

**Table 3:** The differences in the DTI-ALPS index between COVID-19 convalescent patients and healthy controls at different time points.

Hemispheres	Follow-up 1 month	Follow-up 3 months	Follow-up 12 months	HCs		t1 /*χ*^2^ 2 /Z1	*p*1	t2 /*χ*^2^ 2 /Z2	*p*2	t3 /*χ*^2^ /Z3	*p*3
Right brain	1.48 ± 0.19	1.50 (1.45, 1.60)	1.49 (1.39, 1.58)	1.42 ± 0.12	1.40 (1.34, 1.53)	1.520	0.134	−3.326	0.000	−1.493	0.135
Left brain	1.49 (1.39, 1.58)	1.57 ± 0.21	1.54 ± 0.18	1.46 ± 0.12	1.40 (1.33, 1.53)	−0.857	0.391	2.443	0.018	1.918	0.060

*p1: Comparison between the HCs group and the Follow-up 1 month group; p2: Comparison between the HCs group and the Follow-up 3 month group; p3: Comparison between the HCs.*

### 3.3 Longitudinal DTI-ALPS index analysis

**Figure 3:**
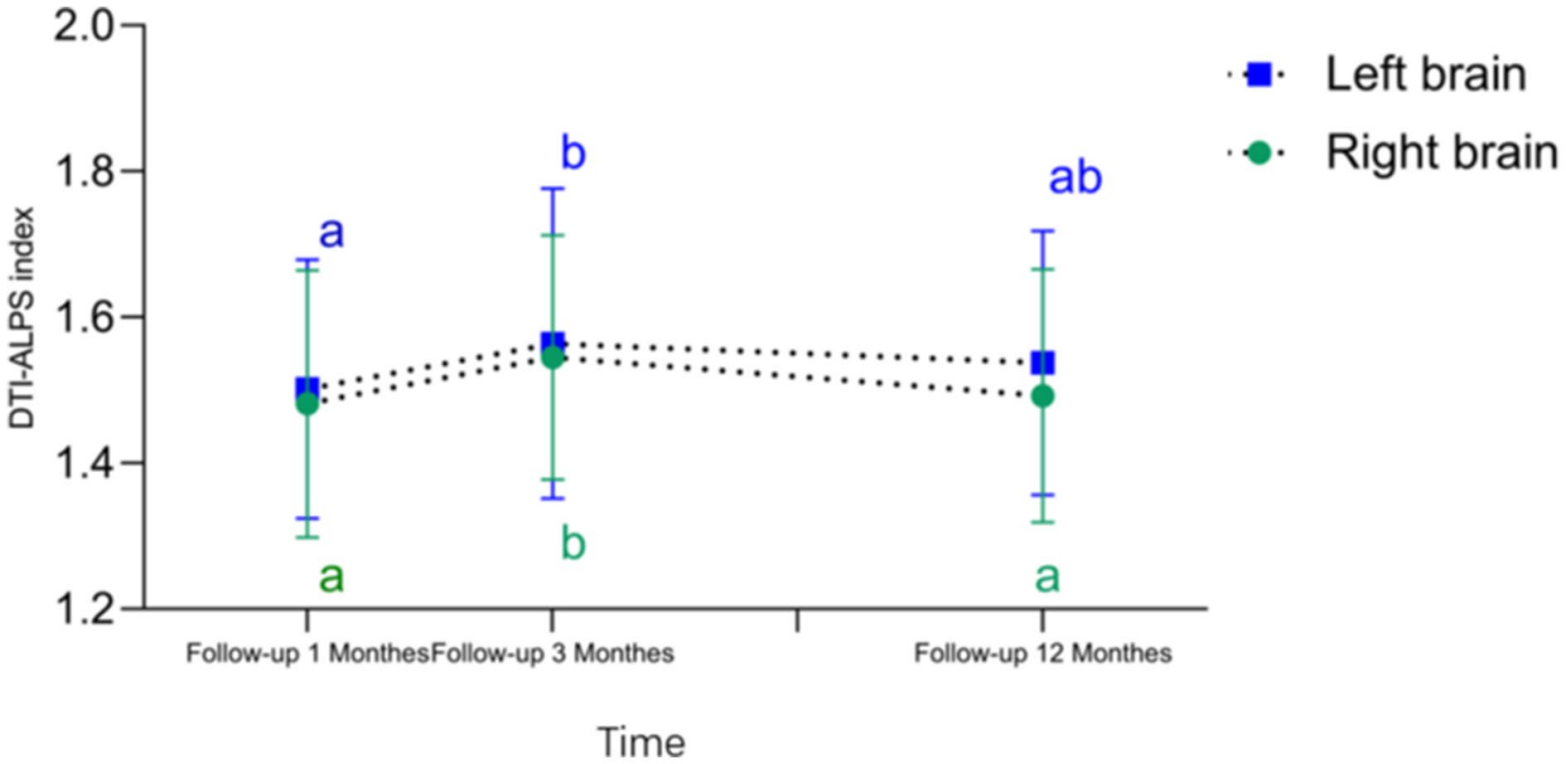
DTI-ALPS index—time curve and differences in DTI-ALPS index across different time periods. Compared to the follow-up 1 months and follow-up12 months, the follow-up 3 months showed an increase in the right brain DTI-ALPS index. Compared to the follow-up1 months, the follow-up 3 months exhibited a significant increase in the left brain DTI-ALPS index (Any common letters indicate no significant difference, while no common letters indicate a significant difference).

As shown in Table 4 and Figure 3, the right-sided DTI-ALPS index was significantly higher in the Follow-up 3 Months group compared to the Follow-up 1 Month and 12-month group. The left-sided DTI-ALPS index was significantly higher in the Follow-up 3 Months group compared to the Follow-up 1 Month group.

**Table 4:** Differences in DTI-ALPS index among recovered COVID-19 patients at 1, 3, and 12 months of follow-up.

Hemispheres	Follow-up 1 month	Follow-up 3 months	Follow-up 12 months	t1//Z1	*p*1
Right brain	1.47 (1.41, 1.56)	1.50 (1.45, 1.60)*	1.49 (1.39, 1.57)◻	8.581	0.014
Left brain	1.49 (1.40, 1.57)	1.52 (1.45, 1.68)^#^	1.52 (1.44, 1.60)	8.537	0.014

Compared with follow-up 1 month, ^*^p < 0.05, ^#^*p* < 0.01; Compared with Follow-up 3 months, ⚪*p* < 0.05, ◻*p* < 0.01.

### 3.4 Correlation analysis

**Figure 4:**
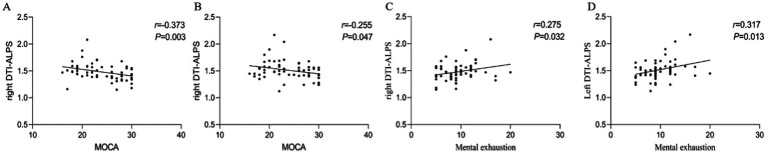
Correlation analysis results. **(A,B)** A positive correlation between bilateral DTI-ALPS index and MOCA scores in patients recovering for 3 months and HC patients. **(C,D)** A positive correlation between bilateral DTI-ALPS indices and mental exhaustion in patients recovering for 3 months and HC patients. DTI-ALPS, diffusion tensor image analysis along the perivascular space; MOCA, Montreal Cognitive Assessment.

The Bilateral brain hemisphere DTI-ALPS index was negatively correlated with the MOCA score (right side: *r* = −0.373, *p* = 0.003; left side: *r* = −0.255, *p* = 0.047). The right-sided and left-sided brain hemisphere DTI-ALPS indices were positively correlated with mental exhaustion (*r* = 0.275, *p* = 0.032; *r* = 0.317, *p* = 0.013, respectively). The DTI-ALPS indices in both brain hemispheres did not show any significant correlation with Correct number of first 3 free recollections or long time delay memory.These results indicate that the changes in the DTI-ALPS index over time were associated with cognitive function and mental exhaustion in the COVID-19 convalescent patients (Figure 4).

## 4 Discussion

we conducted a prospective investigation to explore the longitudinal and cross-sectional changes in the brain lymphatic circulation of COVID-19 patients within 1 year after viral clearance. Our key findings are as follows: (1) Overall cognitive function gradually recovered over time, but showed a significant decline at 3 months after COVID-19 recovery, and mental fatigue persisted even at 1 year; (2) The brain lymphatic circulation in both hemispheres exhibited dynamic changes, gradually increasing at 1 month after COVID-19 clearance and then gradually recovering; (3) The brain lymphatic function was highly active at 3 months after COVID-19 clearance; and (4) The brain lymphatic circulation was associated with overall cognitive function and mental fatigue.

SARS-CoV-2 infection can lead to a variety of neurological features, and fatigue and cognitive impairment are among the most common neurological symptoms of long COVID (Hampshire et al., 2024; Yu et al., 2022). Both our cross-sectional and longitudinal studies revealed abnormal changes in cognitive function and fatigue in COVID-19 convalescent patients. Specifically, the overall cognitive function (assessed by the Montreal Cognitive Assessment, MoCA) showed a significant decline at the 3-month follow-up compared to healthy controls and the 1-month follow-up, but recovered to baseline levels by the 12-month follow-up. Fatigue assessment using the Fatigue Assessment Scale (FAS) indicated that while physical fatigue did not differ significantly from healthy controls across follow-up time points, mental fatigue persisted and remained significantly higher than healthy levels throughout the study period. Previous studies have shown that 40.5% of participants had mild cognitive impairment based on the MoCA score, with declines in auditory-verbal learning and delayed recall (Peskar et al., 2023; Ariza et al., 2024). Moreover, fatigue, cognitive dysfunction (brain fog, memory problems, attention deficits), and sleep disturbances are major features of post-acute COVID-19 syndrome, and neuropsychiatric symptoms may improve over time, but can persist for more than a year (Premraj et al., 2022; Liu et al., 2023). There are also studies that although the overall cognitive impairment in COVID-19 recovered to normal levels within 6 months, some aspects were more severely and persistently impaired, such as attention, concentration, short-term memory, and executive function (Almeria et al., 2024). Within 12 weeks or longer after COVID-19 diagnosis, about one-third of individuals experienced persistent fatigue, and more than one-fifth exhibited cognitive impairment (Alkodaymi et al., 2022).

However, most studies have focused on brain-related symptoms and have not objectively and visually reported on brain lymphatic function, and the potential pathophysiological mechanisms underlying the neurological symptoms in COVID-19 convalescent patients remain to be further explored (Colizzi et al., 2024; Patil et al., 2024). The DTI-ALPS index can provide a non-invasive tool for measuring this in COVID-19 convalescent patients. Some studies using functional MRI (fMRI) or positron emission tomography (PET) have found that recovered COVID-19 patients exhibit alterations in brain functional connectivity and metabolic abnormalities after the acute phase (Guo et al., 2024; Morbelli et al., 2020). Compared to these studies, our findings further suggest that dynamic changes in the brain’s glymphatic system may underlie these functional and metabolic alterations.

The role of lymphatic function in COVID-19 cannot be ignored (Witte and Daley, 2020). SARS-CoV-2 has a certain neuroinvasive ability and can also affect the outcome and function of the brain lymphatic system (Miyasaka, 2022). In the early stage, SARS-CoV-2 may continuously enter the brain parenchyma through the retrograde lymphatic channels within the nasopharyngeal epithelium. The blood–brain barrier function of COVID-19 patients with neurological symptoms may be disrupted without intraparenchymal inflammation (Bhaskar et al., 2020; Mao and Jin, 2020). However, the majority of patients did not show pathological results in cerebrospinal fluid analysis.

Our study found that in the cross-sectional analysis, the DTI-ALPS index of recovered COVID-19 patients was higher in Bilateral cerebral hemispheres at 3 months after viral clearance compared to uninfected participants. In the longitudinal analysis, the DTI-ALPS index at 3 months of follow-up was higher than at 1 month and 12 months of follow-up, with no significant difference between 1 month and 12 months, indicating that the virus’ impact on brain lymphatic circulation was most evident at 3 months, and the brain’s glymphatic system returns to normal after 12 months.Additionally, we observed that some patients exhibited a more pronounced increase in the DTI-ALPS index at the 3-month mark and demonstrated faster recovery by 12 months. In contrast, a few patients showed incomplete recovery of the DTI-ALPS index at 12 months, which may be associated with long-term COVID-19 symptoms, such as brain fog.A subset of patients with long COVID-19 exhibit elevated levels of inflammatory markers (e.g., IL-6) in the blood at 3 months post-infection (Kang et al., 2022), while the activity of lymphatic function is closely associated with inflammatory factors. The brain’s glymphatic function may exhibit a compensatory response to these inflammatory changes. Additionally, SARS-CoV-2 may increase resistance to cerebrospinal fluid (CSF) outflow through the cribriform plate, and patients with post-COVID-19 fatigue syndrome might enhance waste clearance by upregulating glymphatic transport, leading to heightened brain glymphatic activity (Wostyn, 2021). This may be related to the observed increase in the DTI-ALPS index among recovered COVID-19 patients at 3 months post-infection. However, the underlying mechanisms remain unclear and require validation through larger cohort studies and more comprehensive data analysis.

A study of 273,618 COVID-19 patients also showed that 36.55% of individuals developed various clinical symptoms such as fatigue, cognitive impairment, and anxiety between 3 and 6 months after COVID-19 infection, with fatigue (18.82% in 1–180 days; 5.87% in 90–180 days) and cognitive symptoms (7.88% in 1–180 days; 3.95% in 1–180 days) occurring, but not immediately after infection (Taquet et al., 2021). This is consistent with our findings.

Under normal circumstances, the brain parenchyma receives cerebrospinal fluid that flows along the perivascular spaces of arteries, and the interstitial fluid flows out along the perivascular spaces of veins. These two pathways of influx and clearance mainly rely on the communication between the end feet of astrocytes and the gaps between the vessel walls, where the water channel protein AQP4 facilitates the exchange of cerebrospinal fluid and interstitial fluid. The increased DTI-ALPS index suggests enhanced clearance of cerebrospinal fluid through the perivascular spaces of veins (Liu et al., 2021).The lymphatic system’s ability to clear metabolic waste from the brain is widely accepted. During sleep or anesthesia, the interstitial space increases by 60%, leading to a significant increase in the convective exchange of cerebrospinal fluid and interstitial fluid, and the increased convective flow also accelerates the clearance of β-amyloid during sleep (Xie et al., 2013). Brain inflammation and changes in the brain microenvironment can also lead to increased cerebrospinal fluid production (Gumeler et al., 2023; Yu et al., 2022).Certain immune molecules can have neuromodulatory effects on the brain, and the activity of the meningeal lymphatic vessels can alter the accessibility of these neuroimmune modulators in the cerebrospinal fluid to the brain parenchyma, thereby changing their influence on the brain (Xie et al., 2013; Da et al., 2018). This further supports the enhanced cerebrospinal fluid clearance function in COVID-19 convalescent patients. The causes of delayed neurological sequelae might be confirmed by objective brain evidence provided by these MRI scans (Mohammadi and Ghaderi, 2024).

Our study also identified associations between the MOCA score, mental fatigue, and brain lymphatic circulation. The occurrence of cognitive impairment and mental fatigue may be attributed to various factors, such as the accumulation of central toxic metabolites, disruption of the brain microenvironment, and neuronal injury, which can collectively contribute to a series of related neurological diseases and cognitive dysfunction. The lymphatic system plays a crucial role in clearing metabolic waste from the brain and regulating the accessibility of neuroimmune modulators in the cerebrospinal fluid to the brain parenchyma. These functions are essential for alleviating mental fatigue and supporting higher cognitive functions, including learning and memory (Xie et al., 2013; Yu et al., 2024). However, it is important to note that this study design does not establish a causal relationship between these variables. Although our results suggest that impaired glymphatic function is associated with cognitive decline, correlation does not imply causation.

This study has several limitations: 1. Lack of pre-infection and during-infection cognitive, lymphatic, and MRI data: Due to the unanticipated transmissibility of COVID-19 and concerns about nosocomial transmission, the researchers were unable to collect cognitive, lymphatic, and MRI data from the participants prior to and during their SARS-CoV-2 infection. This limits the ability to analyze the complete trajectory from baseline to recovery. Future studies should aim to obtain baseline data on the participants prior to their COVID-19 infection to enable a more comprehensive comparison. 2. Lack of longitudinal comparison with healthy controls: The study only examined the longitudinal changes within the COVID-19 patient group, without a parallel longitudinal comparison with a healthy control group. 3. Larger-scale studies are needed to confirm and expand upon the current results. 4. Lengthy intervals between follow-up assessments: The extended time intervals between the follow-up assessments may have failed to fully capture the long-term temporal evolution of the ALPS index. Despite these limitations, this study provides a novel perspective on the neurobiological underpinnings of cognitive impairment and mental fatigue in COVID-19 convalescent patients, emphasizing the potential role of the brain lymphatic circulation in cognitive recovery. The identified limitations suggest the need for improvements in future research to more comprehensively and accurately unravel this complex issue.

In summary, this prospective study systematically investigated the longitudinal changes in the brain lymphatic circulation of COVID-19 patients within 1 year after viral clearance, and its association with cognitive function and mental fatigue. The results showed that: The overall cognitive function of COVID-19 convalescent patients gradually recovered, but exhibited a significant decline at 3 months post-recovery. However, mental fatigue persisted even at 1 year post-recovery. The brain lymphatic circulation in both cerebral hemispheres exhibited dynamic changes, with the highest level of lymphatic activity observed at 3 months post-recovery. The changes in the brain lymphatic circulation were significantly correlated with the overall cognitive function and mental fatigue, suggesting it may be a potential underlying mechanism for the cognitive impairment and persistent mental fatigue observed in COVID-19 convalescent patients. These findings suggest that enhancing brain glymphatic function, potentially through targeted interventions, may offer a novel therapeutic approach for mitigating long COVID-19 symptoms, including cognitive impairment and mental fatigue, by improving waste clearance and reducing neuroinflammation.

## Data Availability

The original contributions presented in the study are included in the article/supplementary material, further inquiries can be directed to the corresponding author.
